# TIM-1 promotes proliferation and metastasis, and inhibits apoptosis, in cervical cancer through the PI3K/AKT/p53 pathway

**DOI:** 10.1186/s12885-022-09386-7

**Published:** 2022-04-07

**Authors:** Liuyan Chen, Jilin Qing, Yangyang Xiao, Xiaomei Huang, Yanlin Chi, Zhizhong Chen

**Affiliations:** 1 Joint Inspection Center of Precision Medicine, The People’s Hospital of Guangxi Zhuang Autonomous Region & Guangxi Academy of Medical Sciences, Nanning, Guangxi People’s Republic of China; 2 Department of Clinical Laboratory, the first affiliated hospital of Guangxi University of Chinese Medicine, Nanning, Guangxi People’s Republic of China; 3 Center for Reproductive Medicine and Genetics, The People’s Hospital of Guangxi Zhuang Autonomous Region & Guangxi Academy of Medical Sciences, Nanning, Guangxi People’s Republic of China; 4 Department of Clinical Laboratory, Binzhou Medical University Hospital, Binzhou, Shandong People’s Republic of China; 5 Guangxi University of Chinese Medicine, Nanning, Guangxi People’s Republic of China

**Keywords:** T-cell immunoglobulin mucin-1, Apoptosis, Invasion, Metastasis, EMT, Cervical cancer

## Abstract

**Background:**

T-cell immunoglobulin mucin-1 (TIM-1) has been reported to be associated with the biological behavior of several malignant tumors; however, it is not clear whether it has a role in cervical cancer (CC).

**Methods:**

TIM-1 expression in cervical epithelial tumor tissues and cells was detected by immunohistochemistry or real-time quantitative-PCR and western blotting. CC cells from cell lines expressing low levels of TIM-1 were infected with lentiviral vectors encoding TIM-1. Changes in the malignant behavior of CC cells were assessed by CCK-8, wound healing, Transwell migration and invasion assays, and flow cytometry in vitro*;* while a xenograft tumor model was established to analyze the effects of TIM-1 on tumor growth in vivo. Changes in the levels of proteins related to the cell cycle, apoptosis, and Epithelial-mesenchymal transition (EMT) were determined by western blotting.

**Results:**

TIM-1 expression was higher in CC tissues, than in high grade squamous intraepithelial lesion, low grade squamous intraepithelial lesion, or normal cervical tissues, and was also expressed in three CC cell lines. In HeLa and SiHa cells overexpressing TIM-1, proliferation, invasion, and migration increased, while whereas apoptosis was inhibited. Furthermore, TIM-1 downregulated the expression of p53, BAX, and E-cadherin, and increased cyclin D1, Bcl-2, Snail1, N-cadherin, vimentin, MMP-2, and VEGF. PI3K, p-AKT, and mTOR protein levels also increased, while total AKT protein levels remained unchanged.

**Conclusions:**

Our study indicated that TIM-1 overexpression promoted cell migration and invasion, and inhibited cell apoptosis in CC through modulation of the PI3K/AKT/p53 and PI3K/AKT/mTOR signaling pathways, and may be a candidate diagnostic biomarker of this disease.

**Supplementary Information:**

The online version contains supplementary material available at 10.1186/s12885-022-09386-7.

## Background

Cervical cancer (CC) is one of the most frequently diagnosed malignancies in women worldwide, and poses a serious risk to their health [1]. According to data published by GLOBOCAN in 2018, the incidence and mortality of CC rank fourth among female-specific malignant tumors [2]. Most CCs are caused by human papillomavirus (HPV) infection [3]. Moreover, metastasis and recurrence are the main causes of CC-related death, and patients with distant metastasis have poor prognosis [4, 5]. Therefore, it is vital to elucidate the mechanisms underlying the progression and metastasis of CC and to identify new biomarkers and therapeutic targets that can aid in the treatment of this disease.

T-cell immunoglobulin mucin-1 (TIM-1), also known as human kidney injury molecule-1 (KIM-1) and hepatitis A virus cellular receptor 1 (HAVCR1), is a member of the TIM gene family [6]. TIM-1 not only directly enhances the antitumor effect of T cells and NK cells but also alters the tumor microenvironment and induces a more effective antitumor immune response [7, 8]. Furthermore, TIM-1, together with other ligands, can improve the function of immune cells such as Treg cells, Breg cells, dendritic cells (DC), phagocytes, and NK cells, which reduced myeloid-derived suppressor cells (MDSC) in tumor tissues and inhibited tumor growth [7, 9]. Currently, studies investigating diseases and TIM-1 are limited to immunological changes in the microenvironment [7]. However, little is known about its role in cancer. Emerging evidence has suggested that TIM-1 expression is correlated with several aggressive tumors such as renal cell carcinoma [10], human colorectal cancer [11], ovarian clear cell carcinoma [12], hepatocellular carcinoma [13], glioma [14], and gastric cancer [15], highlighting its potential oncogenic role and rendering it a novel and promising target for tumor therapy.

Recent studies have shown that TIM-1 plays an important role in tumor invasion and metastasis. It was shown that depletion of TIM-1 in non-small-cell lung cancer (NSCLC) cell lines A549 and SK-MES-1 could significantly inhibit cell viability, as well as migration and invasion [16]. Xue et al. reported that TIM-1 knockdown inhibited proliferation, colony formation, migration, and invasion of gastric adenocarcinoma cells [17]. Zhou et al. reported that TIM-1 knockdown prevented glioma cell proliferation, invasion, and migration [14]. TIM-1 is highly expressed in gastric cancer cells, and another study showed that TIM-1 knockdown induced apoptosis and inhibited proliferation, migration, invasion, and epithelial-mesenchymal transition (EMT) [18]. Thus, TIM-1 may be directly involved in tumor invasion and metastasis, and may also be related to tumor occurrence and development.

To our knowledge, however, so far as we are aware, no studies have investigated the expression of TIM-1 and its precise biological function in CC. In preliminary experiments, we found that TIM-1 expression was higher in CC tissues, than in high grade squamous intraepithelial lesion (HSIL), low grade squamous intraepithelial lesion (LSIL) and normal cervical tissues. Based on these observations and previous on studies, we hypothesized that TIM-1 could playa role in the occurrence and development of CC, and may be directly involved in the invasion and metastasis of CC cells. To confirm this, we evaluated the expression of TIM-1 in CC and the relationship between TIM-1 expression and the pathological characteristics of patients with CC. Additionally, we also examined the effects of TIM-1 on CC cell proliferation, invasion, and migration, and identified the underlying molecular mechanisms both in vitro and in vivo. Our findings showed for the first time that the effects of up-regulation of TIM-1 were associated with activation of the EMT program and inhibition of apoptosis through modulation of the phosphoinositide 3-kinase (PI3K)/ α-serine/threonine-protein kinase (AKT)/p53 and PI3K/AKT/ mammalian target of rapamycin (mTOR) signaling pathways. Our study provides a novel potential mechanism underlying the development and progression of CC, as well as a promising therapeutic target for the treatment of this disease.

## Materials and methods

### Patient specimens

From 2017 to 2019, cervical biopsy samples were collected from 120 patients at the People’s Hospital of Guangxi Zhuang Autonomous Region who had not received radiotherapy, chemotherapy, or biological therapy prior to surgery. Among the 120 samples, 80 were positive for CC (including 50 cases of squamous cell carcinoma and 30 cases of adenocarcinoma), 30 were positive for cervical intraepithelial neoplasia (CIN) (including 10 cases of CIN I, II, and III cases), and 10 were non-cancerous. Of these, CIN II/III was classified asHSIL and CIN1 as LSIL. In accordance with the Declaration of Helsinki, all patients provided their written informed consent to participate in the study, and the protocols were approved by the Medical Ethics Committee of the People’s Hospital of Guangxi Zhuang Autonomous Region (KY-LW-2020–27).

### Immunohistochemical staining

Immunohistochemical analysis was performed using the Histostain-Streptavidin-Peroxidase Kit (Bioss, Beijing, China) following the manufacturer’s instructions. Insummary, paraffin-embedded normal cervical, CIN, and CC tissue sections were deparaffinized and rehydrated. Antigen retrieval was performed by treating specimens with boiling citrate buffer (pH 6.0) under pressure. Endogenous peroxidase activity was quenched with 3% hydrogen peroxide. The sections were then blocked with goat serum (10%) at room temperature for 20 min to avoid nonspecific staining, and incubated with rabbit anti-human TIM-1 polyclonal antibody (1:300 dilution, ab47635; Abcam, Cambridge, UK) at 4 °C overnight. Subsequently, the sections were incubated with goat anti-rabbit secondary antibody labeled with biotin and horseradish peroxidase (HRP)-labeled streptomycin for 20 min at 37 °C, and then stained with diaminobenzidine and counterstained with hematoxylin. The degree of TIM-1 expression was scored based on the sum of staining intensity (0, no staining; 1, weak staining; 2, moderate staining; and 3, strong staining) and percentage of stained cells (0, 0%; 1, 1%–30%; 2, 31–70%; and 3, > 70%). Finally, the specimens were classified as negative (0), weakly stained (1–2), moderately stained (3), or strongly stained (4–6). Moderate and strong immunoreaction scores were considered high expression; the other final scores were considered low expression.

### Quantitative real-time reverse transcription PCR (RT-qPCR) analysis

Total RNA was extracted from cells using RNAiso Plus (Takara, Dalian, China) and cDNA was synthesized from 1 μg of total RNA using the PrimeScript™ RT Reagent Kit (Takara, Dalian, China) following the manufacturer’s instructions. Quantitative real-time reverse transcription polymerase chain reaction (RT-qPCR) was performed on the ABI 7500 Real-time PCR system (Thermo Fisher Scientific, Waltham, MA, USA) using the TB Green Premix Ex Taq™ II Kit (Takara, Dalian, China). The conditions of the RT-qPCR cycle included an initial denaturation step at 95 °C for 30 s, followed by 40 cycles of 95 °C for 5 s and 60 °C for 34 s. The following primers (Invitrogen; Carlsbad, CA, USA) were used: human *TIM-1*, 5’-AACTGTCTCTACCTTTGTTCCTCC-3’ (sense) and 5’-GTTCTCTCCTTATTGCTCCCTG-3’ (antisense); and glyceraldehyde 3-phosphate dehydrogenase (GAPDH), 5’-ACCCACTCCTCCACCTTTGAC-3’ (sense) and 5’-TCTCTTCCTCTTGTGCTCTTGCT-3’ (antisense). Relative mRNA levels were quantified using the comparative threshold cycle (2^−ΔΔCq^) method with GAPDH as the reference gene [19]. Each assay was performed independently three times.

### Cell lines and culture

The SiHa (Catalogue number TCHu113), C-33 A (Catalogue number TCHu176) cell lines were purchased from the Cell Bank of the Chinese Academy of Sciences (Shanghai, China), and the HeLa (Catalogue number TCHu187) cell line was purchased from the Kunming Cell Bank of the Chinese Academy of Sciences (Kunming, China). Cells from the three lines were cultured in RPMI-1640 medium (Gibco, USA) supplemented with 10% fetal bovine serum (FBS; Gibco, USA), 100 units/mL penicillin, and 100 μg/mL streptomycin (both from Gibco, USA) at 37 °C in the presence of 5% CO_2_.

### Lentiviral (LV) vector construction and cell infection

The LV-TIM-1 homo and LV-negative control (NC) lentiviral vectors were purchased from GenePharma Co. (Suzhou, China). According to the manufacturer’s instructions, the target plasmid (20 μg), vector plasmid pHelper1.0 (15 μg) and the vector plasmid pHelper2.0 (10 μg) were combined evenly with the corresponding volume of transfection reagent (GenePharma Co., China), the total volume was adjusted to 1 mL and incubated at room temperature for 15 min. The mixture was slowly added to the culture medium of 293 T cells (Human Embryonic Kidney Epithelial Cells, Genepharma Co., China) which was less than 12 generations, mixed well, and cultured at 37 °C in an incubator containing 5% CO_2_. The viral supernatant was harvested by ultracentrifugation at 25,000 g at 4 °C and used to transfect the packaging cell line by Lipofectin transfection. Lipofectin transfection was transfected with the transfection reagent prepared by GenePharma Co. and incubated in an incubator containing 5%CO2 at 37 °C for 48 h. HeLa and SiHa cells were infected with lentiviral vectors with a multiplicity of infection (MOI) of 10 for 16 h. Then, the culture medium was removed and fresh medium was added. After 48 h of infection, stably infected cells were selected with 2 μg/mL puromycin (Leagene, Beijing, China). The transfected cells were passaged to at least 5 generations and the infection efficiency was greater than 90%, followed in subsequent experiments. HeLa and SiHa cells were divided into 3 groups: Cells transfected with LV-TIM-1 homo (as the TIM-1 group), transfected with LV-negative control (as the NC group), and non-transfected cells (as the MOCK group).

### In vitro cell proliferation assay

The Cell Counting Kit-8 (CCK-8) and plate colony formation assays were used to evaluate cell proliferation. In the CCK-8 assay, infected HeLa and SiHa cells were plated in 96-well culture plates at a density of 1500 cells per well in triplicate. After incubation for 1–5 days, 10 μL of CCK-8 solution was added to each well. After 2 h of incubation at 37 °C, the absorbance of each well was measured at 450 nm using a Microplate Reader (BioTek, Winooski, VT, USA) and a cell growth curve was drawn. For the colony formation assay, after treatment, cells were plated in 6-well culture plates at a density of 1000 cells per well and cultured for 14 days. Cells were then fixed in methanol and stained with 0.1% crystal violet. Colonies containing more than 10 cells were counted under a microscope. The experiment included the TIM-1 infection group and the MOCK and NC groups, and the experiment was repeated three times.

### Cell cycle assay

The effects of TIM-1 on cell cycle progression were determined by flow cytometry. Propidium iodide (PI) staining was employed to analyze cell cycle phase distribution. The infected cells were collected and fixed in cold 70% ethanol at 4 °C overnight. The cells were then stained for 15 min in PI/RNase staining buffer (BD Biosciences, San Diego, CA, USA). FACScan flow cytometry (BD FACSCanto™ II, BD Biological Sciences, USA) was employed to detect the number and proportion of cells in the G1, G2, and S phases of the cell cycle. The data were expressed as the fraction of cells in the different phases. The experiment included the TIM-1 infection group and the MOCK and NC groups. Each group was examined in triplicate.

### Apoptosis assay

Cells were stained using an Annexin V-APC/PI Apoptosis Detection Kit (KeyGEN BioTECH, China), which could differentiate between intact and apoptotic cells. The cells were then washed twice with cold phosphate-buffered saline (PBS) and resuspended in binding buffer containing 10 μg/mL PI and 10 μg/mL Annexin V-APC. After incubation for 15 min at room temperature in a light-protected area, the specimens were analyzed on a FACScan flow cytometer (BD FACSCanto™ II, BD Biological Sciences, USA) using FlowJo software (Treestar, Inc., USA). The experiment included the TIM-1 infection group and the MOCK and NC groups, and the experiment was repeated three times.

### Wound healing assay

A wound healing assay was performed to assess cell migratory ability. When stably infected HeLa and SiHa cells had reached 80–90% confluence in six-well plates, a wound (scratch) was made in the cell monolayer using a sterile 200-μL pipette tip, followed by washing with starvation medium to remove detached cells. After cultivation for 0, 24, and 48 h, the wounds were imaged at × 100 magnification with a light microscope (Olympus, Japan). The migration rate (MR) was calculated as MR = (initial gap width − final gap width) / initial gap width) × 100%. The experiment included the TIM-1 infection group and the MOCK and NC groups, and each experiment was performed in triplicate.

### Transwell assay

A Transwell assay was used to determine the migratory and invasive abilities of HeLa and SiHa cells. Transwell assays were carried out in 24-well plates using a Transwell chamber (8-μm pore size, Corning, Cambridge, MA, USA) precoated or not with Matrigel (Dilute Matrigel in medium to 1 mg/mL). After being resuspended in serum-free medium, 6 × 10^4^ cells were seeded in the upper chamber, and 600 μL of medium containing 15% FBS was added to the lower Transwell chamber. After culturing for 20–24 h (to assess migratory ability) or 30–36 h (to assess invasive ability), the upper surfaces of the Transwells were wiped with cotton swabs. Cells in the Transwell chamber were fixed in methanol for 30 min, stained with 0.1% crystal violet for 20 min, and then washed with PBS. Next, the number of migrating and invading cells were counted and imaged under a microscope (magnification: × 200; Olympus, Japan) in five random fields per well. The experiment included the TIM-1 infection group and the MOCK and NC groups, and the experiment was repeated three times.

### Xenograft experiments in nude mice

Nine female BALB/c nude mice (4–5 weeks old) were purchased from the Animal Center of Guangxi University of Chinese Medicine (Nanning, China) and raised under specific-pathogen-free conditions, and maintained at 23 ± 3 °C, 60–75% humidity, with a controlled 12 h light–dark cycle, lights on at 07.00 and off at 19.00. Mice were randomly divided into a MOCK group (*n* = 3), a NC group (*n* = 3), and a TIM-1 group (*n* = 3), and subcutaneously injected with SiHa cells (2 × 10^6^ cells in 200 μL of serum-free medium) into the axilla, to establish tumors. Tumor volumes, animal health and behavior were measured every three days. When the tumors had reached an average volume of approximately 1000 mm^3^ (24 days after injection), the mice were anesthetized withan intraperitoneal injection of 50 μL of 20 mg/kg of pentobarbital sodium. One minute later, they were transferred into the cage of the IVIS Lumina LT in vivo Imaging System (PerkinElmer, USA) for monitoring. The axillae of the mice were completely exposed, following which image calibration and visualization were performed using Living Image 4.5 software (PerkinElmer). At the end of the experiment, 150 μL of 20 mg/kg of pentobarbital sodium was injected intraperitoneally for euthanasia. The mouse’s heart and breathing stopped, the pupils dilated, and the tumors were excised and weighed. Tumor volume was calculated using the equation V = A × B^2^ / 2; where A is the largest diameter of the tumor and B the smallest). The animal experiments lasted for a month. All experimental animal protocols used in this study were approved by the Institutional Animal Ethics Committee of Guangxi University of Chinese Medicine (DW20200120-071) and Welfare Committee and followed the guidelines of laboratory animal care in the Declaration of Helsinki.

### Western blotting

The protein extracts were separated using sodium dodecyl sulfate–polyacrylamide gel electrophoresis and transferred to polyvinylidene difluoride (PVDF) membranes (Millipore, Billerica, MA, USA). The membranes were subsequently blocked in 5% skimmed milk or bovine serum albumin (for phosphorylated protein), and incubated with primary antibodies overnight at 4 °C. The primary antibodies used were anti-GAPDH (1:1000, bs-2188R, Bioss), anti-TIM-1 (1:1000, mab1750, R&D Systems, Minneapolis, MN, USA), anti-PI3K (1:1000, C33D4, Cell Signaling Technology, Danvers, MA, USA), anti-AKT (1:1000, C67E7, Cell Signaling Technology), anti-phosphorylated AKT (p-AKT) (1:1000, Ser473, Cell Signaling Technology), anti-mTOR (1:1000, 7C10, Cell Signaling Technology), anti-E-cadherin (1:5000, 20,874–1-AP, Proteintech, Wuhan, China), anti-N-cadherin (1:5000, 22,018–1-AP, Proteintech), anti-vimentin (1:1000, 10,366–1-AP, Proteintech), anti-Snail1 (1:500, 13,099–1-AP, Proteintech), anti-vascular endothelial growth factor (VEGF) (1:500, 19,003–1-AP, Proteintech), anti-matrix metalloproteinase 2 (MMP-2) (1:500, D8N9Y, Cell Signaling Technology), anti-cyclin D1 (1:1000, 92G2, Cell Signaling Technology), anti-p53 (1:1000, 10,442–1-AP, Proteintech), anti-Bcl-2 (1:500, 12,789–1-AP, Proteintech), and anti-BAX (1:5000, 50,599–2-Ig, Proteintech). The next day, the membranes were washed three times and then incubated with horseradish peroxidase (HRP)-conjugated goat anti-rabbit secondary antibody (1:10,000, ab97051, Abcam) and anti-mouse IgG HRP-linked secondary antibody (1:5000, 7076S, Cell Signaling Technology) for 2 h. Finally, the protein bands were detected by enhanced chemiluminescence (Meilun, Dalian, China). Images were captured using the Odyssey Fc Imaging System (LI-COR Biosciences, Lincoln, NE, USA). Protein bands were quantified with ImageJ software (NIH, Bethesda, MA, USA) using GAPDH as the internal control. All experiments were performed in triplicate.

### Statistical analysis

Data are presented as the mean ± standard deviation (SD) for the indicated number of independently conducted experiments. GraphPad Prism 8 (GraphPad Software, San Diego, CA, USA) and SPSS 21.0 (IBM Corp., Armonk, NY, USA) software were used for statistical analysis. The *χ*^*2*^ test was used to analyze the relationship between the clinicopathological parameters of cervical cancer and Spearman’s analysis was used for the correlation analysis. A one-way analysis of variance was used to compare the mean of multiple groups, and the LSD-t test was used for pairwise comparison between groups. Two-sided tests were used for statistical analysis, and the test level *α* = 0.05. *P*-values < 0.05 indicated a statistically significant difference.

## Results

### TIM-1 expression was higher in CC tissues, than in CIN and normal cervical tissues

**Fig. 1:**
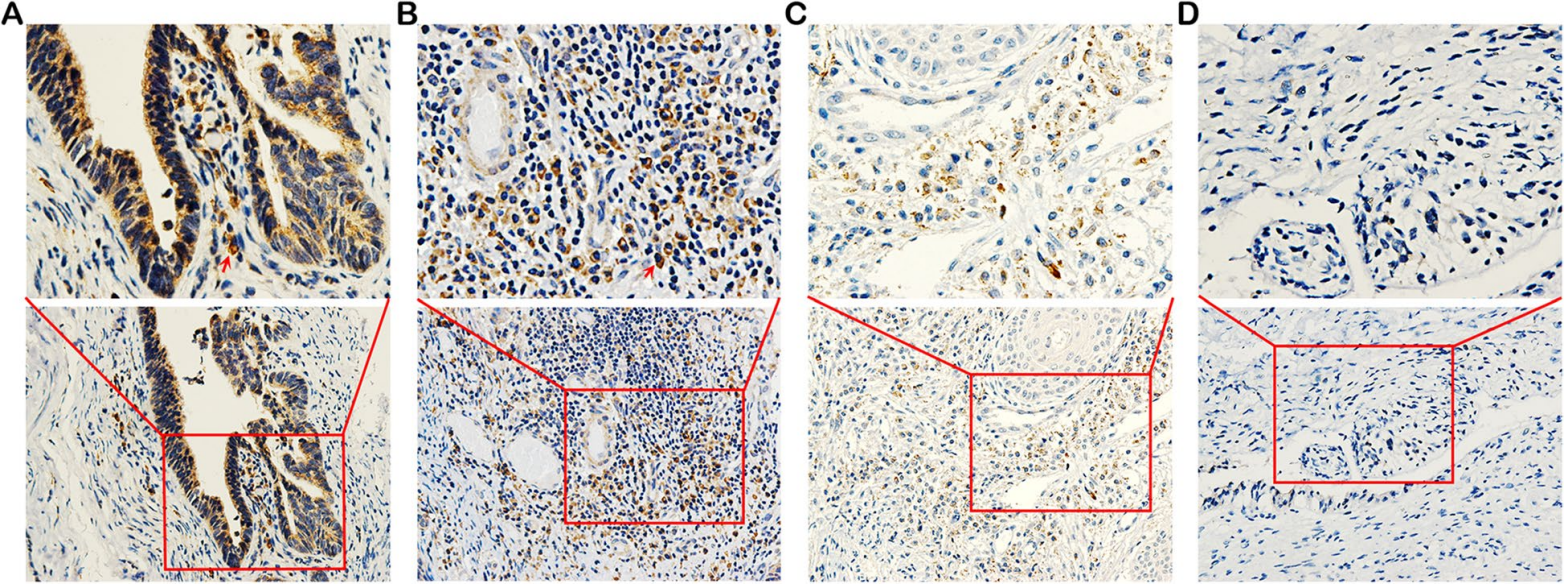
Immunohistochemical staining of TIM-1 expression in cervical tissues. **A** Strong expression of the TIM-1 protein in cervical adenocarcinoma tissue. **B** Moderate expression of the TIM-1 protein in cervical squamous cell carcinoma tissue. **C** Weak expression of the TIM-1 protein in cervical intraepithelial neoplasia tissue. **D** The absence of TIM-1 expression in normal cervical tissue. Lower panels, × 200 magnification; upper panels, × 400 magnification. The red arrowheads indicate positive staining of tumor cells (shown in brown). TIM-1, T-cell immunoglobulin mucin-1

**Fig. 2:**
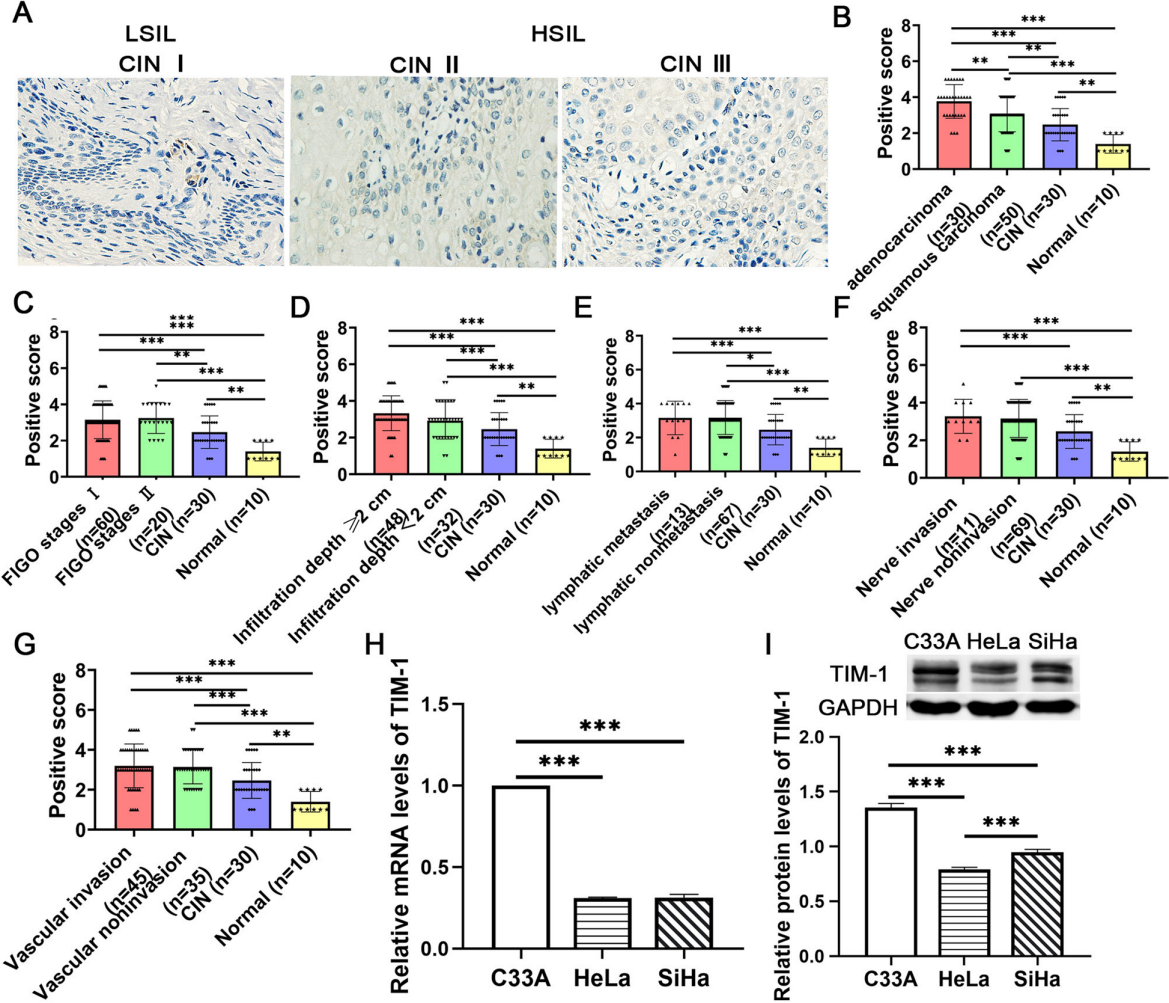
The expression of TIM-1 in cervical tissues and CC cell lines. **A** The expression of the TIM-1 protein in CIN I, II, and III, respectively. **B** The expression level of TIM-1 in normal cervical, CIN, adenocarcinoma and squamous carinoma. **C** The expression level of TIM-1 in normal cervical, CIN, FIGO stages I and FIGO stages II. **D** The expression level of TIM-1 in normal cervical, CIN, Infiltration depth ≥ 1/2 and Infiltration depth < 1/2. **E** The expression level of TIM-1 in normal cervical, CIN, lymph metastasis and lymph nonmetastasis. **F** The expression level of TIM-1 in normal cervical, CIN, nerve invasion and nerve noninvasion. **G** The expression of TIM-1 in normal cervical, CIN, vascular invasion and vascular noninvasion.RT-qPCR (**H**) and western blotting analysis (**I**) of the expression of TIM-1 in three CC cell lines (C-33A, Hela, and SiHa). Data are shown as means ± SD. **P < 0.01 and ***P < 0.001. TIM-1, T-cell immunoglobulin mucin-1; CIN, cervical intraepithelial neoplasia; CC, cervical cancer; HSIL, high grade squamous intraepithelial lesion; LSIL, low grade squamous intraepithelial lesion

Previous studies have suggested that TIM-1 can be considered as an effective biomarker related to tumor development and progression[7]. However, the definite function of TIM-1 in CC is poorly understood. To examine the clinical significance of TIM-1 in human CC, we evaluated the expression of the TIM-1 protein in cervical biopsy samples by immunohistochemistry. The results indicated that the TIM-1 protein was widely distributed in the cytoplasm of tumor cells (Fig. 1A, B), showing strong expression in cervical adenocarcinoma (Fig. 1A) and fairly strong expression in squamous cell carcinoma tissues (Fig. 1B). In some CIN samples, we also detected weak expression of TIM-1 in diseased cells (Fig. 1C), however, there were no significant differences in the expression of LSIL (CIN I) and HSIL (CIN II and III) (*P* = 0.684, Fig. 2A). The TIM-1 protein was not detected in normal cervical epithelial cells (Fig. 1D). As shown in Table 1, TIM-1-positive staining accounted for 81.25% (65/80), 45% (9/20), 30% (3/10), and 0% (0/10) of CC, HSIL, LSIL, and normal cervical tissue sections, respectively. Furthermore, as shown in Fig. 2B, the expression of the TIM-1 protein was significantly higher in the CC tissue than in the CIN or normal cervical tissue (*P* < 0.001), and was higher in the CIN tissue than in normal cervical tissue (*P* < 0.01), and there were no significant differences between the HSIL and LSIL tissue (*P* = 0.509). In conclusion, TIM-1 expression in cervical cancer tissues was up-regulated, which showed that TIM-1 could be a potential biomarker.

**Table 1:** Expression of TIM-1 in cervical tissues

Group	n	Expression of TIM-1			
		**high**	**low**	**χ**^**2**^** value**	***P*-value**
**normal**	10	0	0	39.634	^*^0.000
**LSIL (CIN I)**	10	3	7		
**HSIL (CIN II + III)**	20	9	11		
**CC**	80	65	15		

^*^P < 0.05. *TIM-1* T-cell immunoglobulin mucin-1, *CIN* cervical intraepithelial neoplasia, *CC* cervical cancer, *HSIL* high grade squamous intraepithelial lesion, *LSIL* low grade squamous intraepithelial lesion

We also investigated the relationship between TIM-1 expression and clinicopathological characteristics of patients such as age, histology classification, differentiation level, FIGO stage, tumor diameter, depth of infiltration, involvement of vaginal and lymph node metastases, nerve invasion, and vascular invasion. Our results showed that the expression of TIM-1in cervical cancer tissues was related to the histological classification (Fig. 2B), FIGO stage (Fig. 2C), depth of infiltration (Fig. 2D), lymph node metastasis (Fig. 2E), nerve invasion (Fig. 2F), or vascular invasion (Fig. 2G) compared to CIN tissues(*P* < 0.05) or normal cervical tissues(*P* < 0.05). However, there was no significant difference in TIM-1 expression with age, histology classification, degree of differentiation, FIGO stage, tumor diameter, depth of infiltration, lymph node metastasis, nerve invasion, or vascular invasion between cervical cancer cases (Supplementary Table 1), which may be limited by the small number of samples, and may need to be further confirmed by large samples in future studies. We also found a negative correlation between TIM-1 expression and vaginal involvement (*r* = -0.303, *P* = 0.006, Supplementary Table 1). Collectively, the results showed that TIM-1 may be involved in the progression of CC.

### Expression of TIM-1 in human CC cell lines and lentivirus infection

To investigate the effect of TIM-1 on CC cell lines (C-33 A, HeLa, and SiHa), we first determined the expression of TIM-1 using RT-qPCR and western blotting. The results showed that the levels of TIM-1 expression in the three cervical cancer cell lines were low, but the expression of TIM-1 mRNA (Fig. 2H) and protein (Fig. 2I) in HeLa and SiHa cells was relatively lower than in C-33 A cells. However, the Ct values in RT-qPCR of the three cervical cancer lines were greater than 30 (data not shown), which were not suitable for knockout experiments. Therefore, HeLa and SiHa cells were selected to stably overexpress TIM-1.

**Fig. 3:**
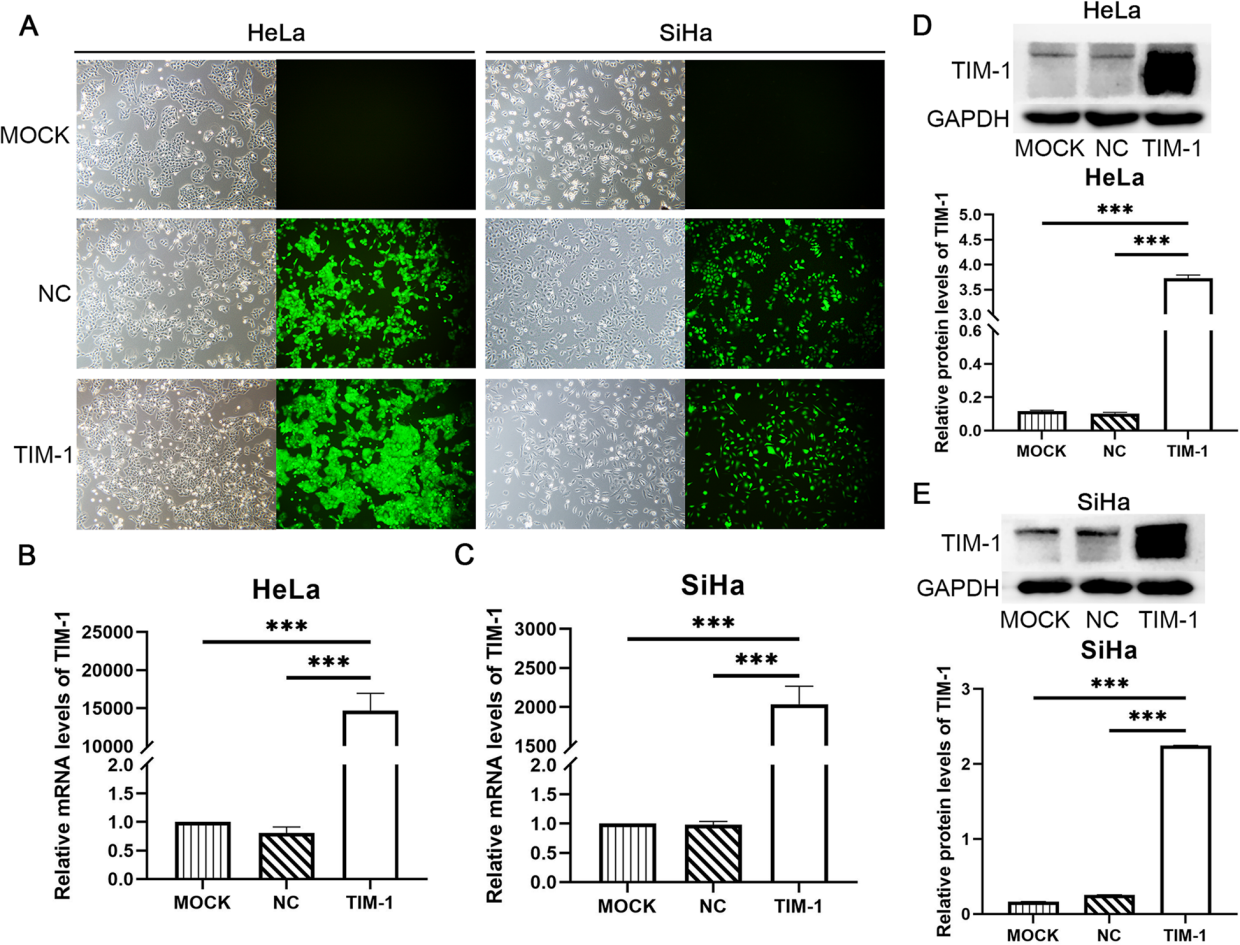
The overexpression of TIM-1 in HeLa and SiHa cells. **A** Transfected HeLa and SiHa cells were monitored under a fluorescence microscope. HeLa and SiHa cells transfected with—lentivirus expressing TIM-1 were analyzed by RT-qPCR (**B**, **C**) and western blotting (**D**, **E**). GAPDH was used for normalization. Data are shown as means ± SD.***P < 0.001. TIM-1, T-cell immunoglobulin mucin-1

We then overexpressed TIM-1 in HeLa and SiHa cells by infecting a lentiviral vector containing full-length TIM-1 (TIM-1 group), and also infected cells with an empty lentiviral vector (NC group). Uninfected cells served as a blank control (MOCK group). After puromycin screening, infected HeLa and SiHa cells were monitored under a fluorescence microscope, which showed that the infection efficiency was greater than 90% (Fig. 3A). TIM-1 mRNA (Fig. 3B, C) and protein (Fig. 3D, E) levels increased significantly in the TIM-1 group (*P* < 0.001) compared to the MOCK and NC groups, while no significant differences were observed between the MOCK and NC groups. Successful overexpression of TIM-1 in HeLa and SiHa cell lines was confirmed by RT-qPCR and western blotting, respectively. Cells from these MOCK, NC, and TIM-1 groups were used for subsequent experiments.

### Overexpression of TIM-1 promoted cell proliferation in vivo and in vitro

**Fig. 4:**
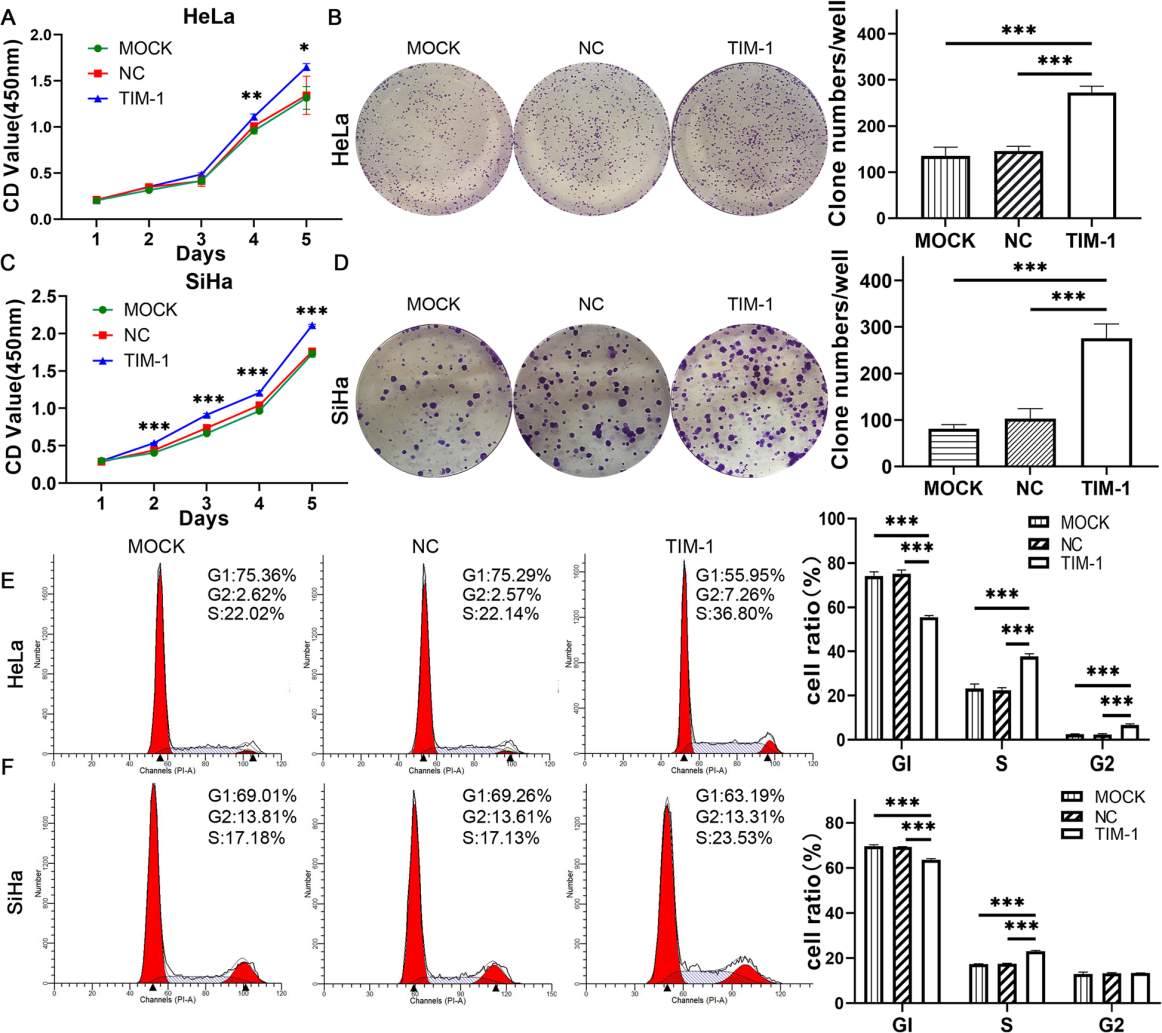
The overexpression of TIM-1 promoted proliferation of CC cells in vitro. Comparison of the proliferation of Hela and SiHa cells transfected with either TIM-1-expressing or NC lentiviral vectors by CCK‐8 assay (**A**, **B**) and (**C**, **D**) clone formation assay. **E**, **F** Comparison of the cell cycle phase distribution of Hela and SiHa cells transfected with either TIM-1-expressing or NC lentiviral vectors by flow cytometry. Untransfected cells served as a blank control (MOCK group). Data are shown as means ± SD. *P < 0.05, **P < 0.01, ***P < 0.001. TIM-1, T-cell immunoglobulin mucin-1; CC, cervical cancer; MOCK, blank control; NC, negative control; CCK‐8, cell counting kit-8

To study the relationship between TIM-1 overexpression and CC development, we next selected HeLa and SiHa cells for the experiments. First explored whether TIM-1 could influence CC cell proliferation in vitro, we performed CCK-8 and colony formation assays. Our results showed that the viability of HeLa and SiHa cells infected with TIM-1 increased compared to that of the MOCK and NC groups (*P* < 0.05), and there were no significant differences between the latter two groups (Fig. 4A, C). Similarly, in the colony formation assay, the number of colonies increased in the TIM-1 group (*P* < 0.001); no significant differences were observed between the MOCK and NC groups (Fig. 4B, D). We then used flow cytometry for cell cycle analysis, and found that the results were consistent with those of the CCK-8 and colony formation assays. When TIM-1 was overexpressed in HeLa cells, there was a significant increase in cell viability and the proportion of cells in the S and G2 phases of the cell cycle, while the number of cells in the G1-phase decreased significantly (*P* < 0.001); no significant differences were observed between the MOCK and NC groups (Fig. 4E). The results also showed that there was a lower percentage of G1-phase SiHa cells in the TIM-1 group than in the MOCK and NC groups, while the percentage of cells in the S phase increased (*P* < 0.001); there were no significant differences between the MOCK and NC groups (Fig. 4F). Combined, these data revealed that TIM-1 promoted the transition of the S/G2-phase cell-cycle in HeLa and SiHa cells.

To explore the molecular mechanisms underlying the effects of TIM-1 on the cell cycle, we measured the protein expression levels of the cell cycle-related markers, cyclin D1 and p53. The results showed that the decrease in the percentage of cells in the G1 phase was accompanied by a decrease in the level of p53 and an increase in that of cyclin D1. There was no significant difference in the protein levels between the MOCK and NC groups (Fig. 6E, F). This indicated that cell viability was markedly increased in the TIM-1 group compared to that in the MOCK and NC groups. Altogether, these data demonstrated that TIM-1 could promote CC cell proliferation through the p53/cyclin D1 signaling pathway in vitro.

**Fig. 5:**
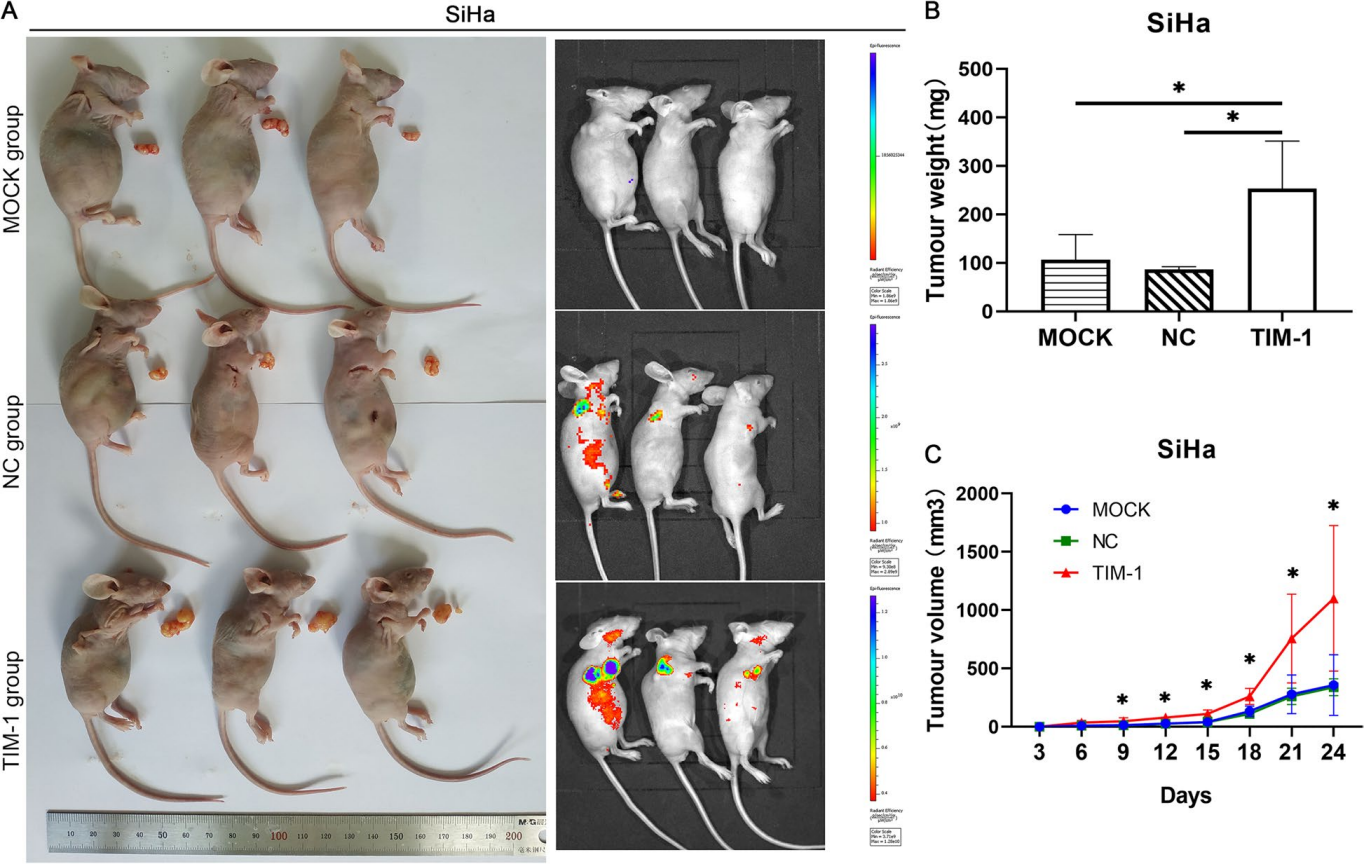
The overexpression of TIM-1 accelerated tumor growth in vivo. **A** Nude mice subcutaneously implanted with SiHa cells were imaged in vivo. Images of three groups of nude mice and tumors were obtained. **B** The tumor weight of mice after euthanasia. **C** Growth curves of xenograft tumors. Tumor size was measured every 3 days. Data are shown as means ± SD. *P < 0.05 *vs*. the MOCK and NC groups. TIM-1, T-cell immunoglobulin mucin-1; MOCK, blank control; NC, negative control

Based on the above findings, we speculated that TIM-1 overexpression could promote tumor growth in animals. To explore this, we next established a xenograft tumor model by injecting infected SiHa cells into nude mice to investigate whether TIM-1 exerts similar growth-promoting effects in vivo. As shown in Fig. 5, the overexpression of TIM-1 promoted xenograft tumor growth in nude mice. Tumor weight (*P* < 0.05) and volume (*P* < 0.05) both increased in the TIM-1 group compared with those in the MOCK and NC groups, while no significant differences were recorded between the MOCK and NC groups (Fig. 5B, C). Collectively, TIM-1 overexpression in CC cells significantly promoted tumor growth, suggesting that TIM-1 plays a proliferative role in the growth of CC cells.

### TIM-1 overexpression inhibited CC Cell apoptosis and modulated the expression of cell apoptosis regulatory molecules

**Fig. 6:**
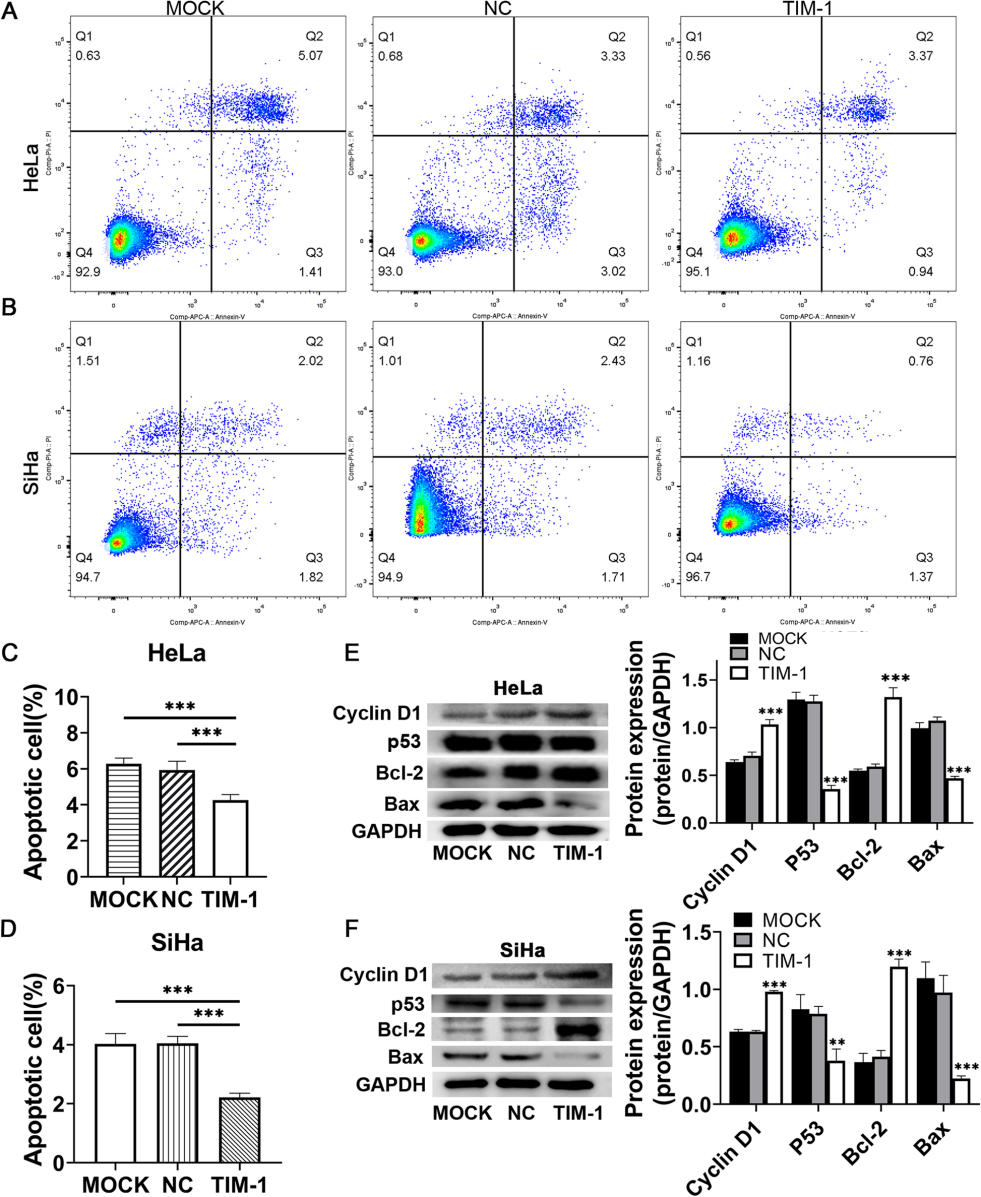
The overexpression of TIM-1 inhibited CC cell apoptosis and modulated the levels of cell cycle- and cell apoptosis-related proteins. The percentages of apoptotic and necrotic HeLa (**A**, **C**) and SiHa (**B**, **D**) cells were determined by flow cytometry. **E**, **F** Western blotting and statistical results of differences in protein levels of cyclin D1, p53, Bcl-2, and BAX. The data represent the averages of three independent experiments. *P < 0.05, **P < 0.01, ***P < 0.001 *vs*. the MOCK and NC groups. TIM-1, T-cell immunoglobulin mucin-1; CC, cervical cancer; MOCK, blank control; NC, negative control

Apoptosis plays an important role in both carcinogenesis and cancer treatment [20]. The effects of TIM-1 on CC cell apoptosis were then evaluated by flow cytometry following Annexin V-APC/PI staining. The results showed that the proportion of apoptotic cells in TIM-1-overexpressing HeLa (Fig. 6A, C) and SiHa (Fig. 6B, D) cells were lower than in the MOCK and NC groups (*P* < 0.001), while there were no differences in the levels of apoptosis between the MOCK and NC groups. Both cyclin D1 and p53 can regulate the expression of a variety of apoptosis-related genes, including *Bcl-2* and *BAX *[21]. To investigate the molecular mechanism underlying the role of TIM-1 in cell apoptosis, we evaluated the expression of the apoptosis marker proteins Bcl-2 and BAX. We found that BAX protein levels were down-regulated in both HeLa and SiHa cells infected with TIM-1, while Bcl-2 protein levels showed the opposite tendency; there was no significant difference in these proteins levels between the MOCK and NC groups (Fig. 6E, F). These results showed that the antiapoptotic effect of TIM-1 overexpression in CC cells may be exerted through downregulation of BAX and upregulation of Bcl-2 protein expression. Furthermore, these data indicated that up-regulation of TIM-1 expression may inhibit cell apoptosis by regulating p53 activation in CC.

### TIM-1 overexpression accelerates migratory and invasive capacity of CC cells in vitro

**Fig. 7:**
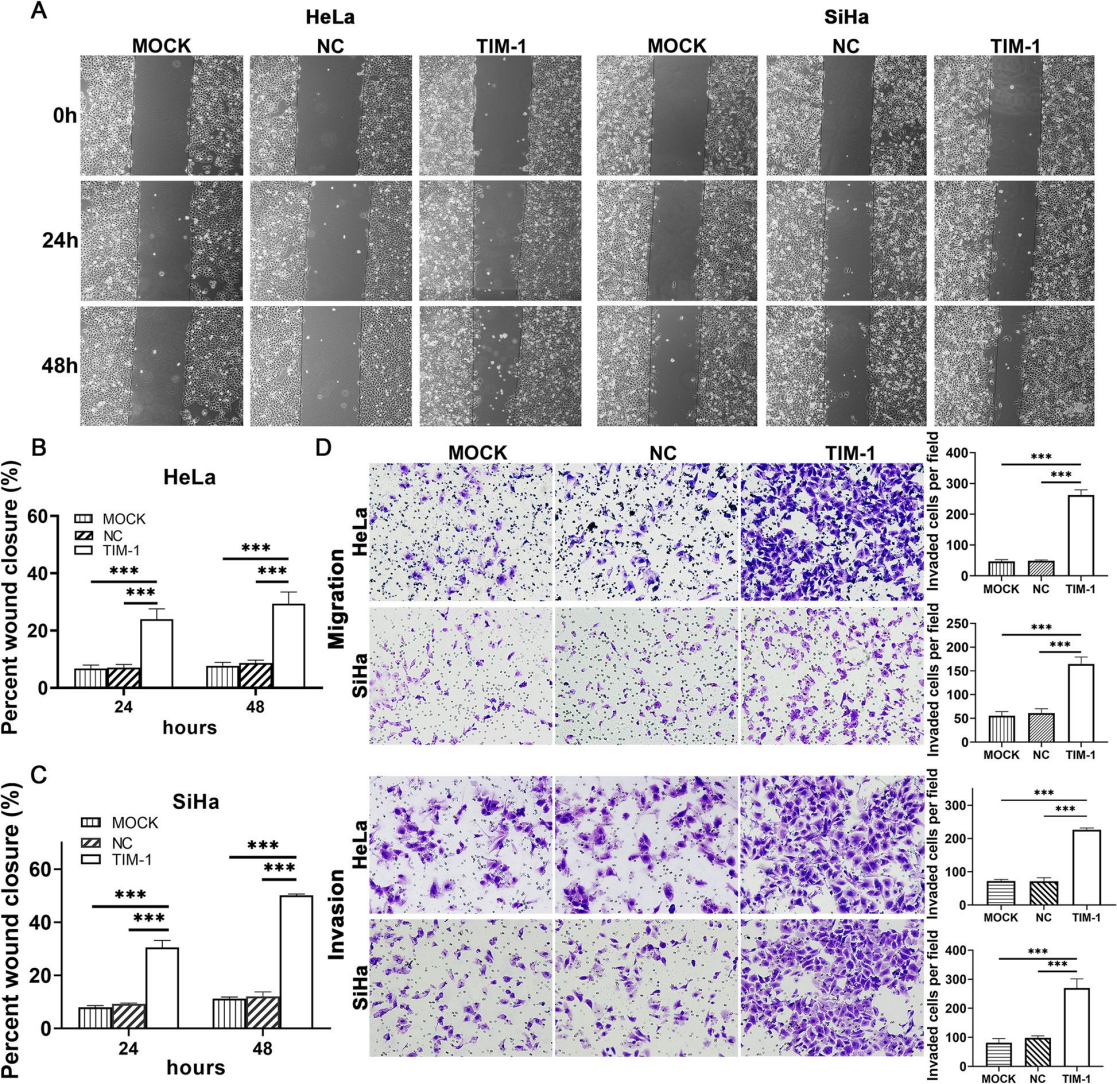
TIM-1overexpression promoted migration and invasion of HeLa and SiHa cells. **A** Microscopic examination diagram of HeLa and SiHa cells in each group at 0, 24, and 48 h after the scratch test (× 100). **B**, **C** The distance migrated by HeLa and SiHa cells in each group at 24 and 48 h after the scratch test. **D** Transwell assays were performed to measure the effects of TIM-1 upregulation on the migratory and invasive abilities of HeLa and SiHa cells. Data are shown as means ± SD. ***P < 0.001. TIM-1, T-cell immunoglobulin mucin-1

Overexpression of TIM-1 promoted the proliferation and growth of CC cells. Therefore, we speculated that TIM-1 might also have an impact on the migration and invasion of CC. To determine whether TIM-1 can influence the metastatic ability of CC cells, we performed wound healing and Transwell migration and invasion assays on infected HeLa and SiHa cells. The results of the wound healing assay showed that the migration rate of HeLa and SiHa cells infected with TIM-1 was significantly higher than in the MOCK and NC groups; no significant differences were observed between the MOCK and NC groups (Fig. 7A–C). Additionally, the Transwell migration and invasion assays showed that cells in the TIM-1 groups had significantly greater migratory potential than cells in the MOCK and NC groups (*P* < 0.001), while there were no significant differences between the latter two groups (Fig. 7D). These results showed that TIM-1 can enhance the migratory and invasive abilities of CC cells.

### TIM-1 induced the expression of EMT-related molecules in HeLa and SiHa cells and promoted their migration and invasion

**Fig. 8:**
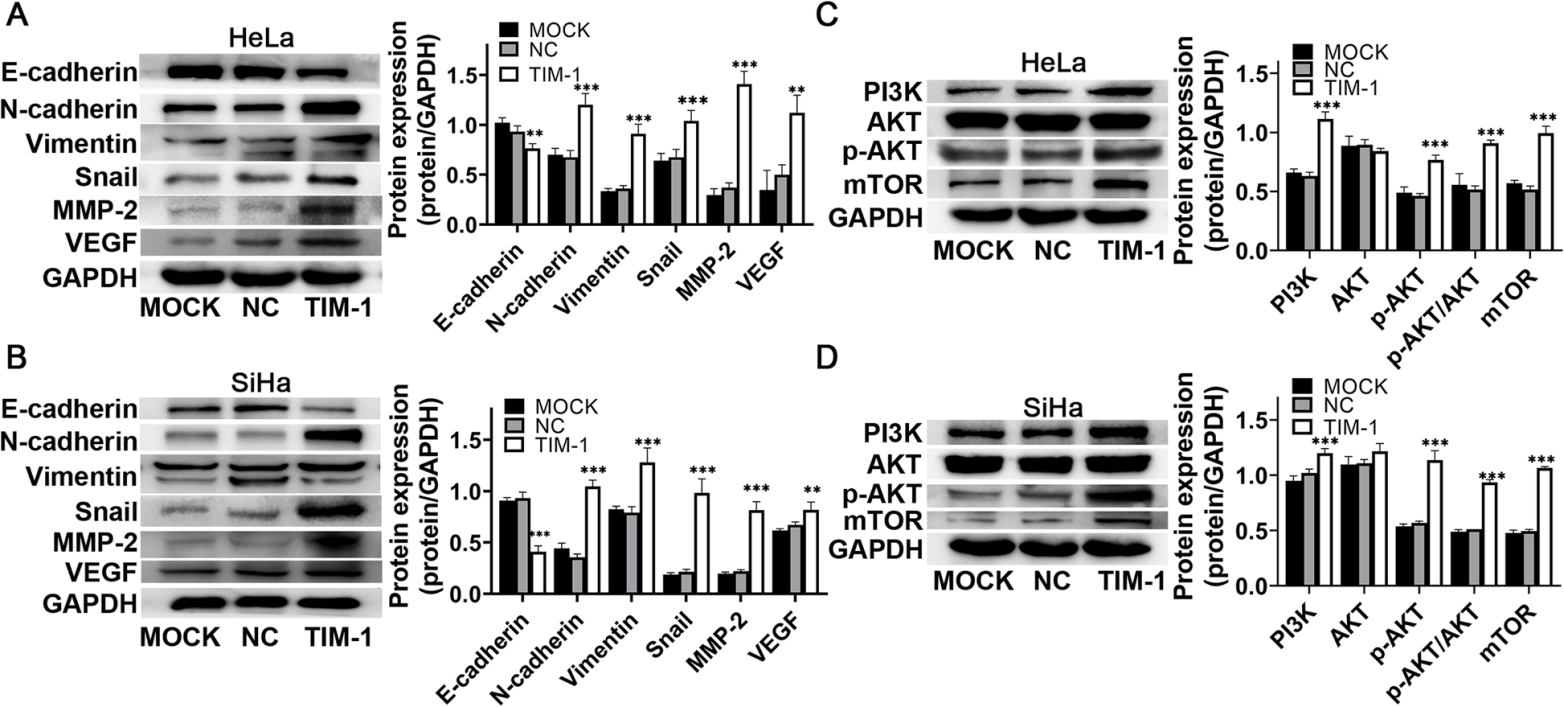
Effect of TIM-1 overexpression on proteins related to the EMT- and PI3K/AKT/mTOR signaling pathway in HeLa and SiHa cells. **A**, **B** Western blotting and quantification analysis of the EMT-associated proteins E-cadherin, N-cadherin, vimentin, Snail, MMP-2, and VEGF. **C**, **D** Western blotting and quantification analysis of proteins related to the PI3K/AKT/mTOR signaling pathway PI3K, p110α, AKT, p-AKT, and mTOR. Data are shown as means ± SD. *P < 0.05, **P < 0.01, ***P < 0.001 *vs*. the MOCK and NC groups. TIM-1, T-cell immunoglobulin mucin-1; EMT, epithelial-mesenchymal transition; MMP-2, matrix metalloproteinase 2; VEGF, vascular endothelial growth factor; MOCK, blank control; NC, negative control

It is well known that EMT significantly increases cancer cell motility and invasiveness [22]. To further explore the effects of TIM-1 on the expression of migration, invasion, and EMT-related proteins, western blotting assays were performed. As shown in Fig. 8A and B, compared to the MOCK and NC groups, the expression of the Snail1, N-cadherin, vimentin, MMP-2, and VEGF proteins increased in the TIM-1 group, whereas that of E-cadherin decreased; while there were no significant differences in the expression levels of these proteins between the MOCK and NC groups. Taken together, these results indicated that TIM-1 can promote the migration and invasion of HeLa and SiHa cells via EMT-related pathways; we reasoned that TIM-1 could play a significant role in CC EMT in vitro.

### TIM-1 promoted CC progression and metastasis through the PI3K/AKT/p53 and PI3K/AKT/mTOR signaling pathway

PI3K/AKT signaling is now identified as a potential target for the treatment of metastatic tumors by mediating the EMT process [23]. Furthermore, the previous studies have shown that TIM-1-mediated T cell stimulation of can recruit the p85 adaptor subunit of PI3K, thus promoting T cell activation through the PI3K pathway [24]. Therefore, we next explored whether the oncogenic effects of TIM-1 were dependent on the PI3K/AKT/mTOR signaling pathway in CC. Our results demonstrated that the expression levels of PI3K, p-AKT, and mTOR proteins were higher in the TIM-1 group than in the MOCK and NC groups, while there were no significant differences in these protein levels between the MOCK and NC groups (Fig. 8C, D). We also observed that TIM-1 could significantly promote AKT phosphorylation without affecting AKT expression in CC cells. This suggests that the PI3K/AKT signaling pathway participates in the progression of TIM-1 mediated CC cells and may act as the downstream modulator of TIM-1. The PI3K/AKT pathway has recently been reported to inhibit the transcriptional activity of p53 as well as its pro-apoptotic functions [25, 26]. Our data also demonstrated that TIM-1 can promote CC cell proliferation via the p53/cyclin D1 signaling pathway in vitro. Altogether, these data revealed that high levels of TIM-1 expression can activate the PI3K/AKT/p53 and PI3K/AKT/mTOR signaling pathway in CC cells, which promoted tumor progression and metastasis.

## Discussion

CC is a commonly diagnosed malignant gynecological tumor. Globally, CC ranks second only to breast cancer in morbidity, while ranking first in incidence and mortality among females from regions with lower human development index [2]. CC has a high potential for prevention, early detection, and cure [27]. The dual prevention strategies of HPV vaccination and CC screening have reduced CC-related incidence and mortality; nevertheless, its incidence still ranks fourth among female-specific cancers in less developed regions of the world [28–30]. HPV-16 and HPV-18 together account for 70–75% of all CCs and 40–60% of CC precursor lesions [31]. Although CC is preventable and curable in the very early stages of the disease, invasion and metastasis remain the main reasons for failure and death of treatment among patients with CC [32]. Therefore, understanding the cellular and molecular mechanisms underlying CC-associated immune modulation is a prerequisite for the development of immunotherapy-based approaches to treat this deadly malignancy.

TIM-1 is preferentially expressed in Th2 cells, where it induces T cell activation and inhibits the development of peripheral tolerance [9, 33]. The expression of TIM-1 can convert a normal epithelial cell into a phagocyte, thus facilitating autophagy and the removal of apoptotic and necrotic cells [34, 35]. In cancer, the apoptotic program is compromised, leading to cell overgrowth and tumor formation [36]. TIM-1 has been linked to immunomodulation, as polymorphisms in the human *TIM-1* gene have been associated with various disorders, including autoimmune diseases, allergies, malignant tumors, and viral infections [7, 33]. Moreover, emerging evidence has shown that TIM-1 participates in the invasion and metastasis of tumor cells, and is involved in the occurrence and development of a variety of cancers [14, 18]. However, little is known about the biological roles of TIM-1 in CC.

In the present study, we found that TIM-1 is a mediator of the progression of CC. Furthermore, we discovered that TIM-1 plays a critical role in the malignant behavior of CC cells, as well as in the occurrence and development of CC. First, our study showed that TIM-1 expression is higher in CC tissues than that in CIN and normal cervical tissues, and is negatively correlated with vaginal involvement. The immunohistochemistry findings indicated that TIM-1 expression in CC tissues was markedly up-regulated compared to in CIN and normal cervical tissues. Our results also showed that TIM-1 expression in cervical cancer tissues was related to histological classification, FIGO stage, depth of infiltration, lymph node metastasis, nerve invasion, or vascular invasion compared to CIN tissues or normal cervical tissues. However, we found that the expression of TIM-1 in CC tissues showed no correlation with age, histology classification, degree of differentiation, FIGO stage, tumor diameter, depth of infiltration, lymph node metastasis, nerve invasion, or vascular invasion in CC cases; and there were no significant differences between HSIL and LSIL. This may have been due tothe small number of samples, and these findings may need to be further confirmed by large samples in future studies.

We also measured TIM-1 mRNA and protein levels in CC cell lines with different HPV phenotypes, and found that TIM-1 expression was low in HeLa (HPV-18-positive) and SiHa (HPV-16-positive) cells, and relatively high in C-33 A cells (HPV-negative). It should be noted that TIM-1 is expressed in three isoforms, and the intracellular TIM-1 protein may be secreted into the extracellular domain, indicating that intracellular protein levels do not increase as significantly as mRNA levels [37].

We also provided evidence that TIM-1 can promote the proliferation of CC cells, both in vitro and in vivo. CCK-8 and colony formation assays revealed that TIM-1 overexpression can greatly enhance the proliferative capacity of CC cells. Furthermore, we found that there were different mitotic abilities, and a reduction in the G1 population and an increase in the S population in both HeLa and SiHa cells overexpressing TIM-1, while an increase in the G2/M population was observed in HeLa cells overexpressing TIM-1. The role of p53 in the regulation of cell-cycle progression through the G1/S phase is well documented [38, 39]. Cyclins are divided into two groups, namely G1/S cyclins, which are essential for the control of the G1-to-S phase transition of the cell cycle and G2/M cyclins, which regulate the G2-to-M-phase transition [40, 41]. Cyclin D1 is a major positive regulator of the G1/S phase transition and therefore also of cell cycle progression, and its dysregulation can lead to abnormal cell growth and angiogenesis, as well as resistance to apoptosis [38, 41, 42]. In this study, the decrease in the proportion of cells in the G1 phase was accompanied by a decrease in p53 levels and an increase in cyclin D1 levels, a major downstream effector of p53 in mediating G1/S phase cell cycle transition [38, 42, 43]. These data suggested that TIM-1-induced cell cycle progression was correlated with down-regulation of p53 and up-regulation of cyclin D1 expression levels. We also showed that TIM-1 promotes CC progression in vivo in a mouse xenograft tumor model. Together, the above results suggest that TIM-1 may regulate CC cell proliferation and cell cycle progression via the p53 signaling pathway.

In addition to enhanced proliferation, resistance to apoptosis is also a hallmark of cancer cells. Apoptosis plays an important role in both carcinogenesis and cancer treatment [20]. We also found that overexpression of TIM-1 reduced the proportions of apoptotic HeLa and SiHa cells. The p53 gene functions as a tumor suppressor through its proapoptotic activities [39, 44]. Generally, the regulation of this form of cell death involves the activity of the p53 and Bcl-2 family genes [20]. The proteins of the Bcl-2 family are known to be important regulators of apoptosis and involve anti- or pro-apoptotic members, such as Bcl-2 and BAX [36, 44]. p53 has been reported to positively regulate BAX expression and negatively regulate Bcl-2 transcription [45]. Our findings demonstrated that up-regulation of TIM-1 significantly decreased the expression of p53 and BAX, and increased that of Bcl-2 and mTOR in CC cells. mTOR is involved in cell growth, proliferation, apoptosis, and many other biological processes mainly through the PI3K/AKT/mTOR signaling pathway [46]. The mTOR pathway also reduces the BAX/Bcl-2 ratio [47]. Taken together, our study showed that p53 and BAX activities were reduced, while those of mTOR and Bcl-2 increased. These results suggest that up-regulation of TIM-1 inhibited CC cell apoptosis through the regulation of p53 and mTOR activation, thus modulating CC pathogenesis and progression.

An increased migratory capability is another prominent characteristic of cancer cells. Wound healing and Transwell migration assays showed that both the migration capacities and migration rates of HeLa and SiHa cells were enhanced with TIM-1 overexpression. MMP-2 belongs to the matrix metalloproteinase family of proteins that can hydrolyze the extracellular matrix and promote invasion of tumor cells [48]. Furthermore, VEGF is the strongest and most specific tumor angiogenesis-promoting factor currently known and can promote tumor angiogenesis, proliferation, and migration [49]. In the present study, we found that the levels of MMP-2 and VEGF were increased in HeLa and SiHa cells that overexpression TIM-1. Taken together, these observations suggest that TIM-1 can enhance the migratory capacity of CC cells.

Tumor cell invasion and metastasis are among the main reasons for the poor prognosis of patients with CC [30]. We found that overexpression of TIM-1 enhanced the invasive ability of CC cells. EMT is closely related to tumor invasion and metastasis [50] and is necessary for tumor cells to leave the site of the primary tumor, invade surrounding tissues, and establish distant metastases. Key effector molecules of EMT include E-cadherin, N-cadherin, and vimentin [22, 50]. N-cadherin and vimentin are two important mesenchymal cell markers and are highly expressed in mesenchymal cells [51]; the loss of E-cadherin is considered a vital event in EMT, a process that is regulated by several transcription factors, including Snail, Slug, and members of the Zeb family [52]. In this study, western blotting revealed that TIM-1 overexpression decreased the expression of E-cadherin while simultaneously increasing that of N-cadherin, vimentin, Snail1, MMP-2, and VEGF. Taken together, our results suggest that TIM-1 can enhance the migratory and invasive abilities of CC cells, as well as induce their EMT by up-regulation of N-cadherin, vimentin, Snail1, MMP-2, and VEGF, and down-regulation of E-cadherin.

The PI3K/AKT/mTOR pathway is known to play a central role in the growth and proliferation of CC cells [43, 53, 54]. Studies have showed that TIM-1-mediated T cell stimulation can recruit the p85 adapter subunit of PI3K, thus promoting T cell activation via the PI3K pathway [24]. Recent, studies showed that TIM-1 knockdown inhibited the biological behaviors of NSCLC and glioma cells through the inactivation of PI3K/Akt pathway [14, 16]. Consequently, we investigated whether the PI3K/AKT/mTOR pathway was involved in the TIM-1-mediated regulation of CC cell functions. Western blotting analysis revealed that the expression levels of PI3K, p-AKT, and mTOR were significantly higher in the TIM-1-overexpressing group than in the MOCK and NC groups, whereas the total AKT protein levels remained unchanged. This demonstrated that TIM-1 can promote the growth and progression of CC by activating the PI3K/AKT pathway, and these effects were positively associated with the expression of cyclin D1, Snail1, N-cadherin, vimentin, Bcl-2, MMP-2, mTOR, and VEGF, and negatively associated with the expression of p53, E-cadherin, and BAX. The PI3K/AKT pathway has recently been reported to inhibit the transcriptional activity of p53 as well as its pro-apoptotic functions [25, 26].

**Fig. 9:**
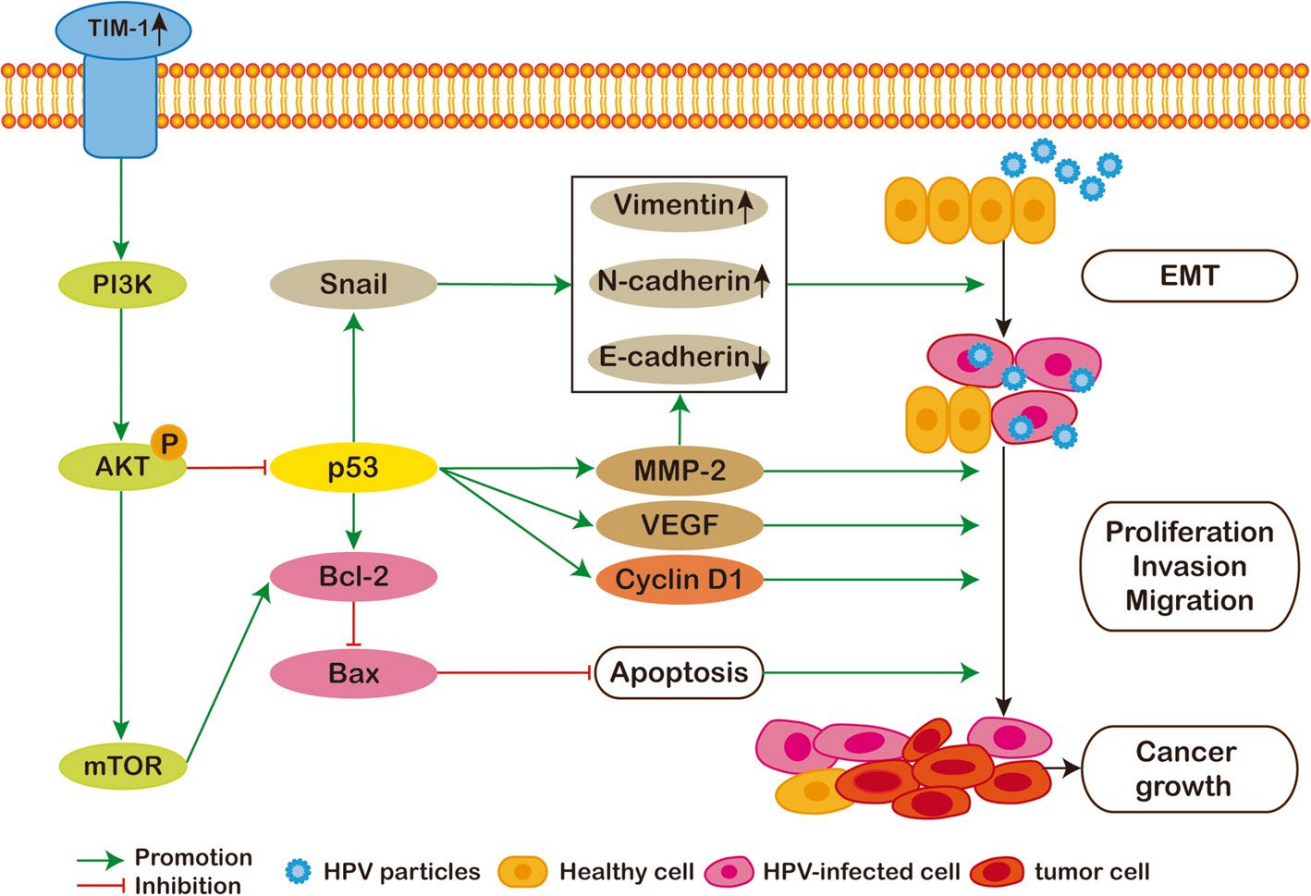
The diagram model showing that TIM-1 overexpression induces epithelial-mesenchymal transition; promotes cell proliferation, migration, and invasion; and inhibits cell apoptosis through the PI3K/AKT/p53 and PI3K/AKT/mTOR signaling pathways in cervical cancer. TIM-1, T-cell immunoglobulin mucin-1; EMT, epithelial-mesenchymal transition; MMP-2, matrix metalloproteinase 2; VEGF, vascular endothelial growth factor; HPV, human papillomavirus

It has been shown that p53 can regulate the expression of MMP-2 [39]. p53 signaling can also affect Snail, Slug, and Twist levels to negatively regulate EMT [39], while loss of function or mutation of p53 has been reported to promote cancer cell EMT by derepressing Snail1 protein expression and activity [55]. Furthermore, there is growing evidence that p53 can negatively regulate VEGF expression [56]. As mentioned above, TIM-1 influenced the p53/cyclin D1-mediated regulation of the CC cell cycle and proliferation, and inhibited apoptosis through its regulatory effects on p53 expression and mTOR activation. Taken together, the results of our study and those of the above-mentioned studies demonstrate that TIM-1 is capable of enhancing the proliferative, invasive, and metastatic potential of CC cells, while also inhibiting their apoptosis, through activation of the PI3K/AKT/p53 and PI3K/AKT/mTOR signaling pathways (Fig. 9).

In conclusion, we provide convincing evidence that TIM-1 expression is markedly increased in CC tissues compared to normal and CIN tissues, and that TIM-1 is also expressed in CC cell lines (C-33 A, HeLa, and SiHa) with different HPV phenotypes. Furthermore, we provided novel evidence that TIM-1 overexpression induced EMT, promoted cell migration and invasion, and inhibited cell apoptosis in CC through modulation of the PI3K/AKT/p53 and PI3K/AKT/mTOR signaling pathways. These results suggest that TIM-1 may play an important role in the occurrence and progression of CC, and may be a valuable biomarker and a potential therapeutic target for CC treatment.

## Supplementary Information

**Additional file 1: Supplementary Table 1** Correlation between TIM-1 expression and clinicopathological characteristics of CC patients.

## Data Availability

The raw data that support the findings of this study are available from the corresponding author upon reasonable request.

## Abbreviations

TIM-1: T-cell immunoglobulin mucin-1
KIM-1: Human kidney injury molecule-1
HAVCR1: Hepatitis A virus cellular receptor 1
CC: Cervical cancer
HPV: Human papillomavirus
CIN: Cervical intraepithelial neoplasia
HSIL: High grade squamous intraepithelial lesion
LSIL: Low grade squamous intraepithelial lesion
MDSC: Myeloid-derived suppressor cells
DC: Dendritic cells
NSCLC: Non-small-cell lung cancer
EMT: Epithelial-mesenchymal transition
PI3K: Phosphoinositide 3-kinase
AKT: α-Serine/threonine-protein kinase
p-AKT: Phosphorylated α-serine/threonine-protein kinase
mTOR: Mammalian target of rapamycin
MMP: Matrix metalloproteinase
VEGF: Vascular endothelial growth factor
FBS: Fetal bovine serum
PBS: Phosphate buffer solution
LV: Lentiviral
NC: Negative control
MOI: Multiplicity of infection
RT-qPCR: Quantitative real-time reverse transcription polymerase chain reaction
GAPDH: Glyceraldehyde 3-phosphate dehydrogenase
CCK-8: Cell counting kit-8
SD: Standard deviation
