# Supplementary material for: TIM-1 promotes proliferation and metastasis, and inhibits apoptosis, in cervical cancer through the PI3K/AKT/p53 pathway
Source: BMC Cancer. 2022 Apr 7;22:370. doi: 10.1186/s12885-022-09386-7 (PMC8991826; doi:10.1186/s12885-022-09386-7)
Supplement: Supplementary file 1 — Additional file 1: Supplementary Table 1 Correlation between TIM-1 expression and clinicopathological characteristics of CC patients. [file 12885_2022_9386_MOESM1_ESM.docx]

**Supplementary table 1: Correlation between TIM-1 expression and clinicopathological characteristics of CC patients.**

| **Characteristics** | **TIM-1 expression** | | **χ^2^ value** | **p value** |
| --- | --- | --- | --- | --- |
|  | **High expression**  **(n=65)** | **Low expression**  **(n=15)** |  |  |
| **Age（years）** |  |  |  |  |
| **≥50** | 38 | 7 | 0.689 | 0.407 |
| **<50** | 27 | 8 |  |  |
| **Histology** |  |  |  |  |
| **Squamous carcinoma** | 38 | 12 | 2.412 | 0.120 |
| **Adenocarcinoma** | 27 | 3 |  |  |
| **Differentiated degree** |  |  |  |  |
| **High** | 17 | 2 | 0.511 | 0.474 |
| **Middle and low** | 48 | 13 |  |  |
| **FIGO stages** |  |  |  |  |
| **Ⅰ** | 48 | 12 | 0.027 | 0.869 |
| **Ⅱ** | 17 | 3 |  |  |
| **Diameter of tumor** |  |  |  |  |
| **≥4cm** | 17 | 4 | 0.000 | 1.000 |
| **<4cm** | 48 | 11 |  |  |
| **Infiltration depth** |  |  |  |  |
| **≥1/2** | 41 | 7 | 1.368 | 0.242 |
| **<1/2** | 24 | 8 |  |  |
| **Involving vaginal stump** |  |  |  |  |
| **Yes** | 5 | 5 | 5.169 | ^*^0.023 |
| **No** | 60 | 10 |  |  |
| **lymphatic metastasis** |  |  |  |  |
| **Yes** | 11 | 2 | 0.000 | 1.000 |
| **No** | 54 | 13 |  |  |
| **Nerve invasion** |  |  |  |  |
| **Yes** | 9 | 2 | 0.000 | 1.000 |
| **No** | 56 | 13 |  |  |
| **Vascular invasion** |  |  |  |  |
| **Yes** | 38 | 7 | 0.689 | 0.407 |
| **No** | 27 | 8 |  |  |
| *P<0.05. TIM-1, T-cell immunoglobulin mucin-1; CC, cervical cancer. | | | | |
